# Biological and prognostic relevance of epigenetic regulatory genes in high-grade gliomas

**DOI:** 10.1093/noajnl/vdae169

**Published:** 2024-10-04

**Authors:** Sonikpreet Aulakh, Joanne Xiu, Andrew Hinton, Sourat Darabi, Michael J Demeure, Soma Sengupta, Santosh Kesari, David M Ashley, Ashley Love Sumrall, Michael J Glantz, David Spetzler

**Affiliations:** West Virginia University, Morgantown, West Virginia, USA; Caris Life Sciences, Medical Affairs, Phoenix, Arizona, USA; Caris Life Sciences, Medical Affairs, Phoenix, Arizona, USA; Hoag Family Cancer Institute, Newport Beach, California, USA; Translational Genomics Research Institute, Phoenix, Arizona, USA; Hoag Family Cancer Institute, Newport Beach, California, USA; School of Medicine, The University of North Carolina at Chapel Hill, Chapel Hill, North Carolina, USA; Pacific Neuroscience Institute and Saint John’s Cancer Institute, Santa Monica, California, USA; Duke University School of Medicine, Durham, North Carolina, USA; Levine Cancer Institute, Charlotte, North Carolina, USA; Departments of Neurosurgery and Oncology, Penn State Hershey Medical Center, Hershey, Pennsylvania, USA (M.J.G.); Caris Life Sciences, Medical Affairs, Phoenix, Arizona, USA

**Keywords:** chromatin remodeling, clinical oncology, glioma, molecular oncology

## Abstract

**Background:**

High-grade gliomas (HGGs) are the most aggressive type of gliomas and have the poorest outcomes. Chromatin remodeling (CR) genes have been implicated in multiple oncogenic pathways in numerous cancer types. In gliomagenesis, CR genes have been implicated in regulating the stemness of glioma cells, the tumor microenvironment (TME), and resistance to therapies.

**Methods:**

We performed molecular profiling of 4244 HGGs and evaluated associations of CR mutations with other cancer-related biomarkers, infiltration by immune cells, and immune gene expression. We also evaluated the association between CR mutations and survival in wild-type *IDH* HGG patients.

**Results:**

Nearly 10% of HGGs carry mutations in CR genes, with a higher prevalence (15%) in HGGs with *IDH* mutations. Analysis of cooccurrence with other biomarkers revealed that CR-mutated HGGs possess favorable genetic alterations which may have prognostic value. CR-mutated HGGs with wild-type *IDH* demonstrated colder TME and worse OS overall compared to the CR-wild-type HGGs.

**Conclusions:**

Our study reveals the prognostic effects of CR mutations in HGG and points to several biomarker candidates that could suggest sensitivity to emerging therapeutic strategies.

## Lay Summary

High-grade gliomas (HGGs) are aggressive brain cancers that sometimes have a mutation in a gene called IDH. Changes in a group of genes related to chromatin remodeling (CR) can influence how tumor cells grow and respond to treatments. The authors of this study wanted to see how often changes in CR genes were found in patients with HGGs. To do this they studied over 4000 patients with HGG. Their results showed that 15% of patients with HGG who have a mutation in IDH, also have mutations in CR genes. Patients who do not have a mutation in IDH have mutations in CR genes less than 10% of the time. Additionally, patients with HGGs who don’t have mutations in IDH but do have mutations in CR genes tend to live for shorter periods of time than those without these changes.

**Key Points**

- Nearly 10% of HGGs carry mutations in chromatin remodeling genes.
- CR-mutated HGGs with wild-type *IDH* possess significantly different genetic alterations and colder tumor microenvironment compared to the wild-type.
- CR-mutated HGGs with wild-type *IDH* are associated with worse OS overall in univariate and multivariate analysis.

**Importance of the Study**

High-grade gliomas (HGGs) are the most aggressive brain tumors with the poorest outcomes, despite aggressive multimodal treatment regimens. Chromatin remodeling (CR) genes have been implicated in multiple aspects of gliomagenesis including resistance to therapies. It is important to further develop our understanding of epigenetic factors to identify potential molecular interactions, gain insights about prognostic opportunities, and help to identify biomarkers that can be exploited with future targeted therapies. The goal of this study was to explore the molecular landscape of HGGs that are associated with mutations in CR genes. We performed molecular profiling of 4244 HGGs and evaluated associations of CR mutations with other cancer-related biomarkers, immune cell infiltration, immune gene expression, as well as survival in univariate and multivariate analyses. Our study points to several biomarker candidates for emerging therapeutic strategies.

Gliomas are the most common tumor of the central nervous system, accounting for about 30% of brain tumors and 80% of malignant brain tumors.^1^ Gliomas are categorized into 4 different grades that are associated closely with tumor growth rates. High-grade glioma (HGGs), including glioblastoma (GBM) and grade 3/4 astrocytoma, are the most aggressive brain tumors and consequently have the poorest outcomes. The recent 2021 WHO classification categorized HGGs according to *IDH*, *EGFR*, and *pTERT* status.^2^ Standard treatment of HGGs involves maximum safe surgical resection, at which time tissue acquisition enables molecular profiling. Surgery is usually followed by a combination of radiotherapy and treatment with the DNA alkylating agent temozolomide (TMZ). Despite this aggressive multimodal treatment regimen, outcomes from HGGs remain poor.^3^

Chromatin remodeling (CR) genes include components of the Switch/Sucrose Non-Fermentable (SWI/SNF) CR complexes and other factors that dynamically regulate epigenetic modification of DNA and histones. Epigenetic regulation of gene expression and genome stability have been implicated in multiple oncogenic pathways in numerous cancer types.^4–6^ Consequently, several epigenetic modifiers have been used to treat cancers with varied success. In gliomagenesis, CR genes have been implicated in inducing stemness of glioma cells,^7^ regulating immune cells within the tumor microenvironment (TME),^8^ and impacting resistance to therapies including TMZ.^8^

It is important to further develop our understanding of epigenetic factors to identify potential molecular interactions, gain insights about prognostic opportunities, and help to identify biomarkers that can be exploited with future targeted therapies. Therefore, the goal of this study was to explore the molecular landscape of HGGs that are associated with mutations in CR genes (CR-mut). We performed molecular profiling of 4244 HGGs and evaluated associations of CR-mut with other cancer-related biomarkers, infiltration by immune cells, and immune gene expression. Finally, we evaluated the association between CR-mut and survival in IDH WT HGG patients.

## Materials and Methods

### Sample Collection From Participants

Two patient cohorts were identified. Cohort one comprised 4244 HGGs including GBM and grade 3/4 astrocytoma (Table 1) that underwent comprehensive tumor profiling at Caris Life Sciences, including whole transcriptome sequencing and whole exome sequencing (WES). Cohort 2 comprised 5527 HGG patients for whom survival data was available from the CODEai database, which integrates molecular data with treatment information and clinical outcomes data. This study was conducted in accordance with the guidelines of the Declaration of Helsinki, the Belmont Report, and the US Common Rule. In keeping with 45 CFR 46.101 (b), this study was performed utilizing retrospective, deidentified clinical data from patients with central nervous system cancer. Therefore, this study was considered Institutional Review Board exempt and no patient consent was necessary from the subjects.

**Table 1.** Patient Characteristics and Prevalence of Chromatin Remodeling Gene Mutations

		Glioblastoma, IDH-WT	Astrocytoma, IDH-MT	*q*-value
Gender	Male	59.2% (2187/3695)	60.5% (332/549)	.567
	Female	40.8% (1508/3695)	39.5% (217/549)	
Age	Median age [range] (*N*)	62 [1–>89] (3695)	38 [12–78] (549)	<.0001
Chromatin remodeling gene mutation		Mutated/total *N*	Mutated/total *N*	
	Chromatin remodeling Gene mutation rate	328/3695 (8.87%)	84/549 (15.30%)	<.0001
	SWI/SNF	110/3695 (3.06%)	54/549 (9.80%)	<.0001
	ARID1A	47/3694 (1.27%)	20/549 (3.64%)	<.0001
	ARID2	31/3685 (0.84%)	13/548 (2.37%)	.014
	SMARCA4	18/3687 (0.49%)	18/546 (3.30%)	<.0001
	PBRM1	20/3695 (0.54%)	4/548 (0.73%)	1
	SMARCB1	9/3695 (0.24%)	3/548 (0.55%)	.76
	SMARCE1	7/3570 (0.20%)	0/530 (0.00%)	1
	Histone methyltransferase	190/3695 (5.14%)	31/549 (5.65%)	.62
	SETD2	138/3675 (3.76%)	16/545 (2.94%)	.975
	KMT2D	25/3577 (0.70%)	11/519 (2.12%)	.046
	KMT2C	23/3649 (0.63%)	3/536 (0.56%)	1
	KMT2A	22/3660 (0.60%)	3/545 (0.55%)	1
	EZH2	10/3695 (0.27%)	1/548 (0.18%)	1
	NSD1	6/3692 (0.16%)	1/548 (0.18%)	1
	Other (DNA methyltransferase, histone acetyltransferase, histone demethylase, transcription coactivator)	82/3693 (2.22%)	14/549 (2.55%)	.628
	DNMT3A	45/3285 (1.37%)	5/478 (1.05%)	1
	EP300	10/3685 (0.27%)	3/548 (0.55%)	.842
	KDM6A	15/3643 (0.41%)	1/537 (0.19%)	1
	KDM5C	2/3141 (0.06%)	1/452 (0.22%)	.975
	ASXL1	23/2931 (0.78%)	4/407 (0.98%)	1

Distribution of CR mutations among the entire cohort (4244 HGG tumors) and mutation frequency of individual CR genes. The mutation percentage was calculated as mutated/(mutated + wild type). Indeterminate results were excluded from the calculations.

### Genomic and Transcriptomic Analysis

NGS was performed on genomic DNA isolated from formalin-fixed paraffin-embedded (FFPE) tumor samples. For analysis on the NextSeq platform (Illumina), a custom-designed SureSelect XT assay was used to enrich 592 whole-gene targets (Agilent Technologies). For NovaSeq analysis (WES), a hybrid pull-down panel of baits designed to enrich for more than 700 clinically relevant genes at high coverage (>500×) and high read-depth was used, along with another panel designed to enrich for an additional >20 000 genes at lower depth (>250×). Prior to molecular testing, tumor enrichment was achieved by harvesting targeted tissue using manual microdissection techniques. Genetic variants identified were interpreted by board-certified molecular geneticists and categorized as pathogenic or likely pathogenic according to the American College of Medical Genetics and Genomics (ACMG) standards. Only pathogenic or likely pathogenic variants were included in the analyses. Matched tumor/normal samples were not analyzed to determine somatic versus germline variants. All variants were detected with >99% confidence based on allele frequency and amplicon coverage, with an average sequencing depth of coverage of >500× and an analytic sensitivity of 5%.

For RNA sequencing (RNA-Seq), FFPE specimens underwent pathology review to evaluate the percent tumor content and tumor size. For tumor enrichment, a minimum of 10% tumor content in the area for microdissection was required to extract RNA. Biotinylated RNA baits were hybridized to the synthesized and purified cDNA targets and the bait-target complexes were amplified in a post-capture PCR reaction. The resultant libraries were quantified and normalized. Then the pooled libraries were denatured, diluted, and sequenced. The reference genome used was GRCh37/hg19 and analytical validation of this test demonstrated ≥97% positive percent agreement, ≥99% negative percent agreement and ≥99% overall percent agreement with a validated comparator method. Transcripts per million values were generated using the Salmon expression pipeline for transcription counting. Immune cell fractions were calculated from the deconvolution of bulk RNA-Seq data using the quanTIseq computational pipeline.^9^

For microsatellite instability (MSI) or microsatellite stable (MSS) status of the tumors, >2800 target microsatellite loci were examined and compared to the reference genome hg19. Tumor mutational burden (TMB) was measured by counting all non-synonymous mutations found per tumor that had not been previously described as germline alterations in dbSNP151, Genome Aggregation Database (gnomAD), or benign variants identified by Caris geneticists. A cutoff point of ≥10 mutations per MB was used to define “high tumor mutational burden” (TMB-high), based on the KEYNOTE-158 pembrolizumab trial.^10^ Caris Life Sciences is a participant in the Friends of Cancer Research TMB Harmonization Project.^11^

### Real-World Evidence

Real-world overall survival (OS) information was obtained from insurance claims data for 5527 HGG tumors previously analyzed by WES or 592-gene-panel of DNA sequencing at Caris. The cohort included samples from HGG patients from sample collection to the last contact. Kaplan–Meier estimates were calculated for molecularly defined patient cohorts using the Cox-proportional regression model for both univariate and multivariate analyses.

### Statistical Analysis

The molecular features of tumors carrying pathogenic or likely pathogenic (P/LP) and CR mutations were compared to CR-WT. Categorical data was assessed using a chi-square or Fisher Exact test, where appropriate. Immune cell abundance in the tumor micro-environment was estimated using the method described above^9^ and significance was tested using a nonparametric Wilcoxon rank-sum test. Gene expression for immune checkpoint genes was normalized to the median gene expression in the control group (CR-WT) and fold change was calculated; significance was tested using nonparametric Wilcoxon rank-sum test. *P*-values were adjusted for multiple hypothesis testing by Bonferroni or Benjamini-Hochberg techniques. All statistical analyses were 2-sided at a significance level set to an adjusted *p*-value, or *q* of .05.

### Availability of Data and Materials

The deidentified sequencing data are owned by Caris Life Sciences. The datasets generated during and analyzed during the current study are available from the authors upon reasonable request and with permission of Caris Life Sciences. Qualified researchers may contact the corresponding author with their request.

## Results

**Figure 1.**
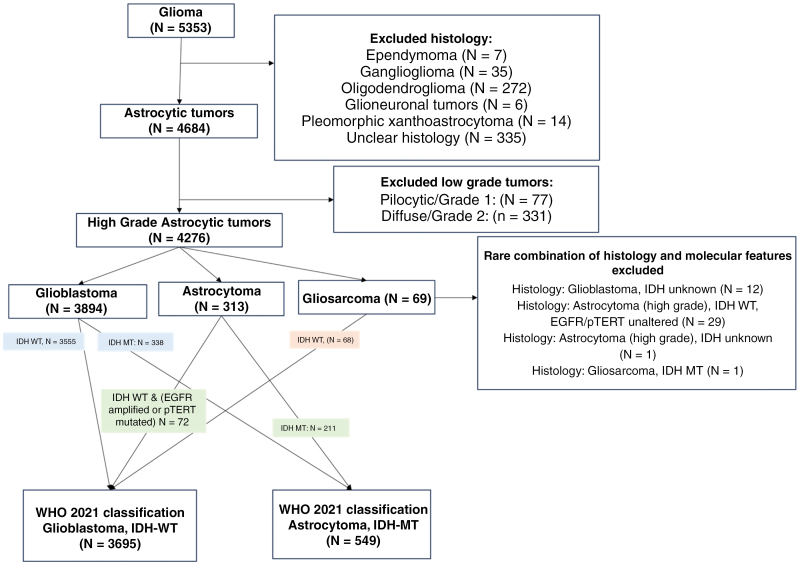
Cohort selection diagram.

From the large real-world database of 5353 glioma tumors that were submitted to comprehensive molecular profiling at Caris Life Sciences, a total of 4276 high-grade tumors with astrocytic histology were retrospectively identified after excluding non-astrocytic and low-grade histologies. Based on the WHO 2021 classification, these high-grade astrocytic tumors were grouped into astrocytoma, *IDH-*MT (*N* = 549), and glioblastoma, *IDH*-WT (*N* = 3695). Rare combinations of histology and molecular features including oligodendroma, histologically noted glioblastoma tumors with unknown *IDH1/2* status, or *IDH-*WT high-grade astrocytic tumors with wild-type *EGFR* or *TERT* promoter mutations were excluded from the analysis (Figure 1). Among these 2 cohorts, 15.3% (84/549) of the astrocytoma, *IDH*-mut and 8.87% (328/3695) of the glioblastoma, *IDH*-WT samples displayed ≥1 mutation of 17 CR genes that were considered (Table 1). Overall, a higher prevalence of CR mutations in the astrocytoma, *IDH*-mut group was seen (15.3% vs. 8.9% in glioblastoma, *IDH*-WT, *q* < .0001), and the difference was primarily found in components of SWI/SNF complexes (9.8% vs. 3.1%, *q* < .0001) and one histone methyltransferase (HM), including *ARID1A* (3.64% vs 1.27%, *q* < .0001), *ARID2* (2.37%, vs 0.84%, *q* = .014), *SMARCA4* (3.30% vs 0.49%, *q* < .0001), and *KMT2D* (2.12% vs 0.70%, *q* = .046) in astrocytoma, *IDH*-mut and glioblastoma, *IDH*-WT, respectively. Two other CR genes with the highest mutation rates were *SETD2* (2.94% vs 3.76%) and *DNMT3A* (1.05% vs 1.37%), which did not differ significantly in prevalence between the astrocytoma and glioblastoma groups.

**Figure 2.**
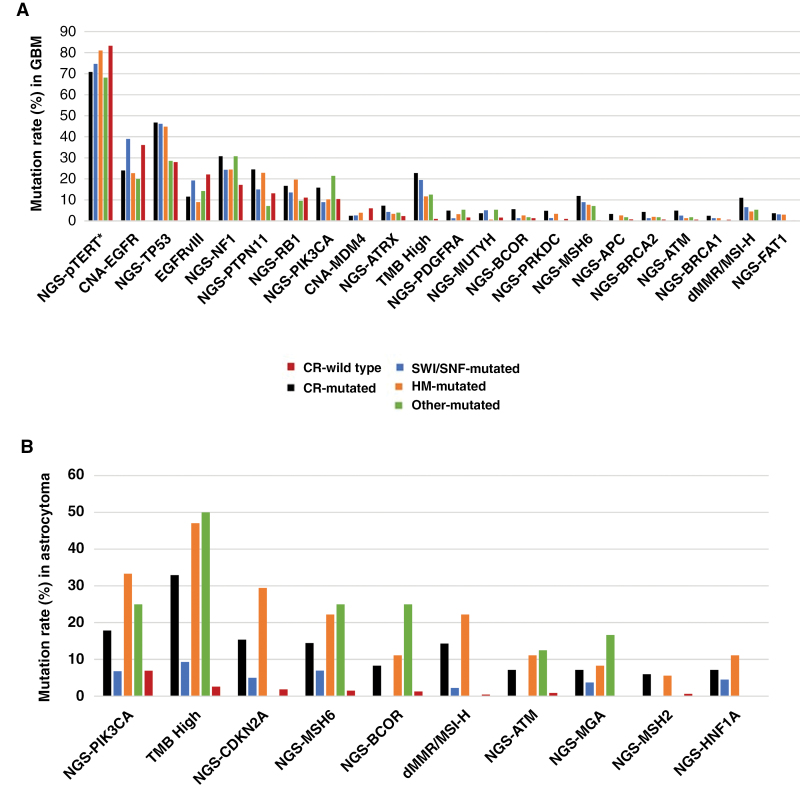
Molecular differences in chromatin remodeling (CR)-mut versus CR-WT high-grade gliomas. (A) Glioblastoma, IDH-WT and (B) Astrocytoma, IDH-mut. Shown are alterations occurring in ≥0.5% of tumors with a *q* < .05 when the CR-mut group for all CR genes is compared to the CR-WT group. Subgroups of the CR-mut cohort are sorted into classes of CR genes: SWI/SNF, histone methyltransferase (HM), and other (DNA methyltransferase, histone acetyltransferase, histone demethylase, transcriptional coactivator). *Due to the high GC-rich region in the TERT promoter, mutation rates in this gene were manually scored.

We compared the molecular landscape of CR-mut tumors with CR-WT in both GBM*, IDH*-WT tumors (Figure 2A), and astrocytoma (Figure 2B). Among statistically significant alterations differing between GBM, *IDH*-WT CR-mut compared to CR-WT, a higher prevalence of TMB-high (22.8% vs. 0.96%) and dMMR/MSI-high (11% vs 0.09%) were noted, which coincides with significantly higher mutation rates in numerous DNA damage repair genes, including *BRCA1* (2.45% vs 0.6%), *BRCA2* (4.3% vs 0.7%), *ATM* (4.9% vs 07%), *MSH6* (11.9% vs 0.4%), and *MUTYH* (3.7% vs 1.6%), suggesting increased DDR deficiency. Interestingly, *MGMT* methylation is not significantly different between the 2 groups (47% vs 40%), while other key GBM-associated alterations including *TERT* promoter mutation (71% vs 83%), *EGFR* amplification (24% vs 36%), and *EGFRvIII* mutation (12% vs 22%), were significantly less prevalent in CR-mut tumors as compared to CR-WT. Substantial variability was seen among the subgroups of CR genes, including a notable cooccurrence of SWI/SNF mutation with *EGFR* amplification (38%) and *EGFRvIII* (19%).

Similarly, comparing CR-mut to CR-WT in astrocytoma, *IDH-*MT disclosed a significantly higher prevalence of TMB-high (33% vs 2.6%) and dMMR/MSI-high (14% vs 0.4%). DDR genes including *MSH6* (14.5% vs 1.5%), *ATM* (7.1% vs 0.9%), and *MSH2* (6.0% vs 0.65%) were more frequently mutated in the CR-mut group. Effects in *TP53* (86% vs 90%), *EGFR* amplification (0 vs 0.6%), *EGFRvIII* (0 vs 0.2%), and *pTERT* (12% vs 8%) were not significant. A significantly increased prevalence of *CDKN2A* mutation (15% vs 0.1%) was seen in the CR-mut astrocytoma group which was not observed in the GBM (5% vs 3%) comparison.

**Figure 3.**
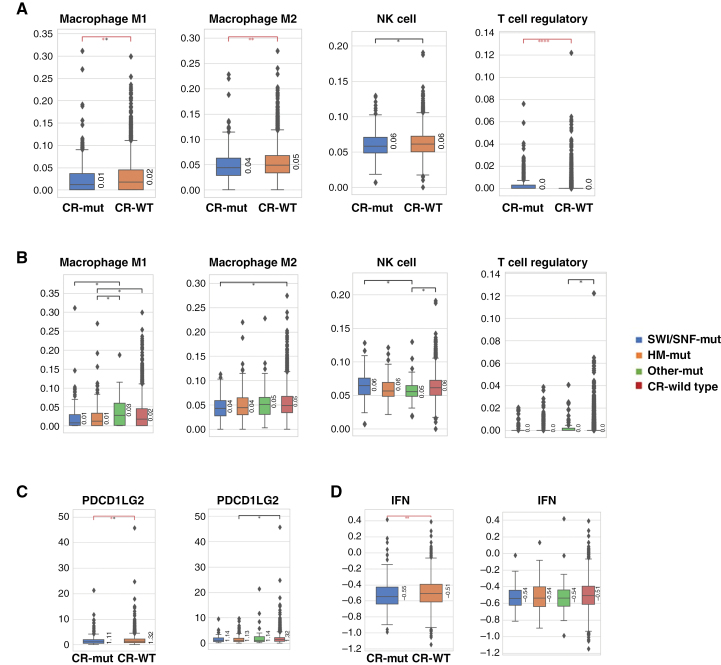
Association of chromatin remodeling (CR) mutations with TME in glioblastoma, IDH-WT. (A) Immune cell populations in CR-WT compared to CR-mut. (B) Immune cell populations in CR-WT compared to classes of classes of CR genes: SWI/SNF, histone methyltransferase (HM), and others. (C) Expression of selected immune-related genes in CR-WT compared to CR-mut and CR-WT compared to CR-mut gene classes. (D) IFN score in CR-WT compared to CR-mut and CR-WT compared to CR-mut gene classes. Brackets and asterisks indicate statistical significance; red and black colors indicate *P*-values and *q*-values, respectively. *<.05; **<.01; ***<.001; and ****<.0001.

**Figure 4.**
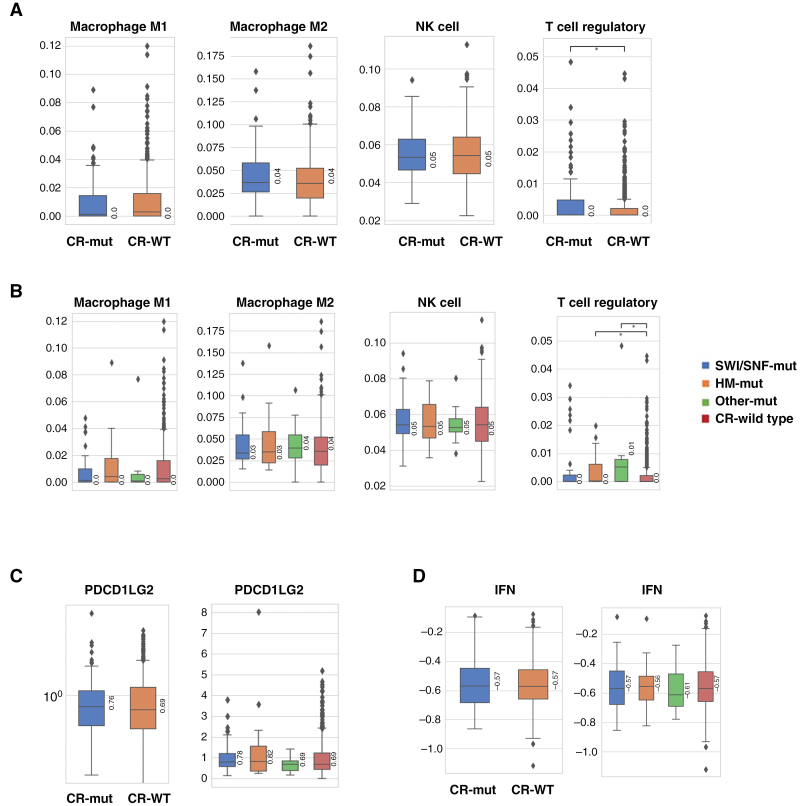
Association of chromatin remodeling (CR) mutations with TME in astrocytoma, IDH-mut. (A) Immune cell populations in CR-WT compared to CR-mut. (B) Immune cell populations in CR-WT compared to classes of CR genes: SWI/SNF, histone methyltransferase (HM), and others. (C) Expression of selected immune-related genes in CR-WT compared to CR-mut and CR-WT compared to CR-mut gene classes. (D) IFN score in CR-WT compared to CR-mut and CR-WT compared to CR-mut gene classes. Brackets and asterisks indicate statistical significance; red and black colors indicate *P*-values and *q*-values, respectively. *<.05; **<.01; ***<.001; and ****<.0001.

In addition to the substantially different genomic landscapes seen in CR-mut vs. CR-WT in both GBM and astrocytoma, we also compared lymphocyte infiltration in the tumor microenvironment (TME) using RNA deconvolution. GBM, *IDH-*WT CR-mut tumors had significantly lower infiltration of M1 macrophage (*q* < .05), M2 macrophage (*q* < .01), and NK cells (*P* < .05) compared to CR-WT tumors, while regulatory T cells (Tregs) were increased (*q* < .0001; Figure 3A). Performing the same analysis in CR-mut subgroups revealed that lower M1 macrophage levels were more highly associated with HM mutations (*P* < .05), M2 macrophage decrease with SWI/SNF mutations (*P* < .05), NK decrease with mutations in “Other” (*q* < .05), and Treg increase with “Other” (*q* < .05; Figure 3B). TME comparison in astrocytoma, *IDH*-mut revealed a much less pronounced difference in CR-mt versus CR-WT tumors, with the only difference seen in a slight enrichment of Treg in CR-mut tumors when compared to CR-WT (*P* < .05; Figure 4A).

Analysis of expression of immune-related genes in GBM, *IDH*-WT revealed significantly lower expression of PD-L2 (*PDCD1LG2*) in CR-mut compared to CR-WT (*q* < .05), which was lower in all 3 CR gene groups but only significantly in HM-mut GBM, *IDH*-WT (*P* < .05; Figure 3C). IFN-signature was also lower in CR-mut compared to CR-WT (*P* < .01) in the GBM, *IDH*-WT cohort (Figure 3D). No significant difference between CR-mut and CR-WT in immune gene expression and IFN score were observed in astrocytoma, *IDH*-mut (Figure 4).

**Figure 5.**
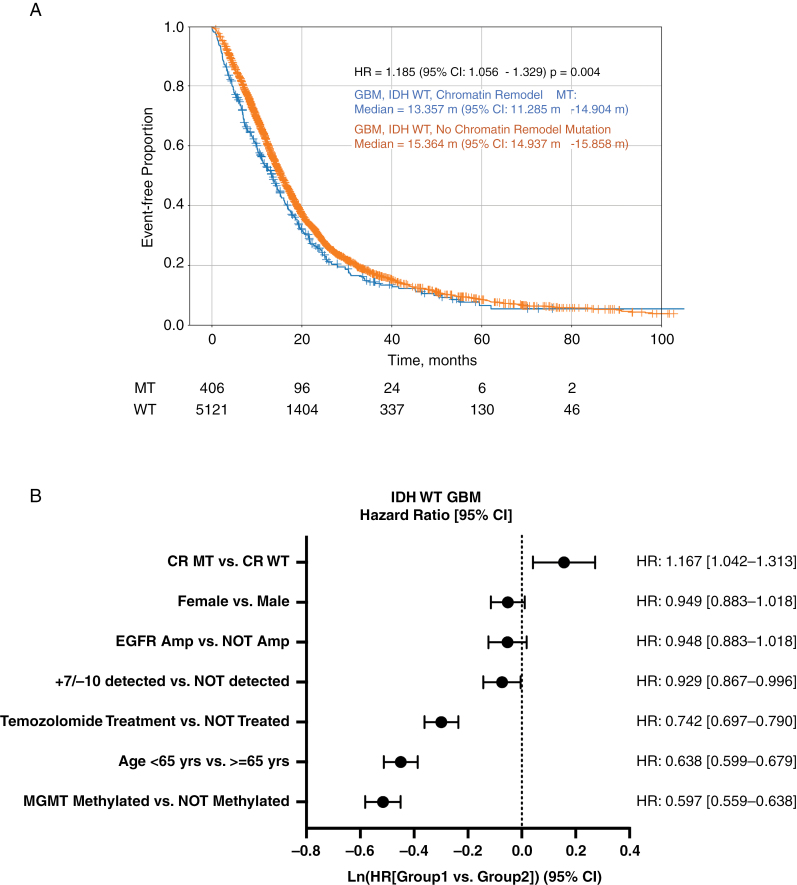
Survival in HGG patients with or without CR mutations. (A) Kaplan–Meier estimate of survival in CR-mut versus CR-WT in glioblastoma, IDH-WT. (B) Multivariate analysis of glioblastoma, IDH-WT with corrections for CR-mutations, gender, EGFR amplification, trisomy 7 and monosomy 10 (+7/−10), TMZ treatment, age, and MGMT methylation status.

To characterize associations of CR mutations with clinical outcomes in GBM and astrocytoma, we used real-world data from a cohort of patients that had been subjected to molecular profiling. Among GBM, *IDH*-WT tumors, CR-mut was associated with worse OS with a median difference of 2.01 months (median of 13.4 months in CR-mut vs 15.4 months in CR-WT, HR: 1.185, 95% CI: 1.056–1.329, *P* = .004; Figure 5A). Among astrocytoma, *IDH-*mut, no significant difference in OS was seen between CR-mut and CR-WT. Multivariate analysis was performed on the GBM, *IDH*-WT cohort to evaluate other well-known factors such as gender, age, Chr +7/−10 (gain of chromosome 7 and loss of chromosome 10), *EGFR* amplification, TMZ treatment, and *MGMT* methylation. This multivariate analysis confirmed a favorable prognosis for CR-WT tumors (HR = 1.167; 95% CI: 1.042–1.313), while also expectedly confirming favorable effects with TMZ treatment (HR = 0.742; 95% CI: 0.697–0.790), younger age (<65 years; HR = 0.638; 95% CI: 0.599–0.679), and *MGMT* methylation (HR = 0.597; 95% CI: 0.559–0.638; Figure 5B). Surprisingly, the effect of Chr +7/−10 was weakly favorable for prognosis. Female gender and *EGFR* amplification trended toward favorable outcomes, but the effect was not significant.

## Discussion

Molecular profiling of a large cohort of HGGs including grade 4 GBM, *IDH*-WT, and grade 3/4 astrocytoma, *IDH*-mut have enabled the exploration of potential associations of CR genes with biomarkers of clinical significance in these malignant astrocytic tumors, yielding some significant observations among subgroups of interest. CR gene mutations occurred in 8.87% of grade 4 GBM, *IDH*-WT, and 15.3% of grade 3/4 astrocytoma, *IDH*-mut. Analysis of cooccurrence with other biomarkers revealed that CR-mut HGGs possess genetic alterations which may have prognostic value. CR-mut GBM and *IDH*-WT tumors also demonstrated colder TME. CR-mut grade 4 GBM, *IDH-*WT showed worse OS compared to CR-WT GBMs.

In the GBM, *IDH-*WT cohort, HM genes represented the highest mutation rates (5.14%). While the astrocytoma, *IDH*-mut exhibited a similar rate of HM mutations (5.65%), a significantly increased rate of SWI/SNF mutations (9.8%) occurred (Table 1). One previous study demonstrated that *SETD2* (HM gene) was associated with lower survival in TMZ-treated GBM patients with methylated MGMT promoters, and played a role in MSH6-mediated TMZ resistance.^12^ However, the same study targeted multiple histone demethylases resulting in restored TMZ sensitivity in *SETD2*-mut cell lines. Increased *MSH6* mutations are also associated with CR-mut tumors in our study, indicating the potential utility of multiple biomarkers in future applications of this type of therapeutic approach.

*ARID1A* (AT-rich interaction domain 1A) was the most commonly mutated gene among SWI/SNF components in both *IDH*-WT (1.27%) and *IDH*-mut (3.64%) cohorts (Table 1). As one of several SWI/SNF genes that have exhibited synthetic lethality, *ARID1A* has come under study for various promising targeting strategies.^13,14^ In the context of genome stability, loss of *ARID1A* function can be compensated by ATR activation, and ATR inhibitors have resulted in lethality in *ARID1A-*deficient cell lines.^15^ *ARID1A*-deficient cells have also exhibited sensitivity to PKL1 inhibition through a separate pathway involving mitochondrial function.^16^ Independently of *ARID1A, PLK1* has also been reported to increase TMZ sensitivity in glioma stem cells and in vivo,^17,18^ suggesting potential in combinatorial approaches. In the context of gliomas, a subset of HGG patients with appropriate genetic profiles may be candidates for clinical trials involving some of these strategies.

TMB-high status and dMMR/MSI-high status were detected in CR-mut tumors at a significantly higher frequency in both *IDH*-WT and *IDH*-mut cohorts. Although they have been associated with SWI/SNF mutations in multiple cancer types,^19,20^ they have not been adopted as predictors of immune checkpoint inhibitor (ICI) response in glioma. *ARID1A* mutations, along with other members of the ARID family (*ARID1B*, *ARID2)*, have been associated with positive predictive value for ICI therapy in multiple cancer types.^21^ However, validation of this approach in GBM and astrocytoma has been limited. In our study, colder TME was apparent in CR-mut HGGs as evidenced by lower infiltration of macrophages in the *IDH*-WT group and higher Tregs in both cohorts (Figures 2 and 3), an observation that has previously been ascribed to gliomas in general. Our observation that significantly lower expression of ICI biomarkers in CR-mut tumors may indicate that this subcohort of HGGs may be even less sensitive to ICI therapy. However, if CR genes play a role in the establishment or maintenance of cold tumor environments, it may also provide future opportunities for targeted therapy approaches. SWI/SNF complexes have come under study recently as targets for therapeutic strategies including ICI.^4,22^ More elucidation of epigenetic mechanisms in gliomagenesis may facilitate the application of some of these strategies in the future.

The molecular landscape of CR-mut in GBM, *IDH*-WT, and astrocytoma, *IDH-*mut tumors include increased mutations in multiple genes of previously demonstrated prognostic or therapeutic value. Among the most frequently occurring mutations in HGGs are *TP53* and *CDKN2A*,^23,24^ which were observed at a higher mutation rate in CR-mut in our study in GBM, *IDH*-WT, and astrocytoma, *IDH*-mut, respectively (Figure 2). Conversely, *EGFR* amplification is associated with poor prognosis in glioma,^25,26^ and was significantly reduced in CR-mut GBM, *IDH-*WT overall (Figure 2). Multiple studies of some of these genes have explored potential targeted therapy strategies.^27^

Interestingly, *ATRX* was mutated at a higher rate in CR-mut tumors (7.2%) compared to CR-WT (2.3%) in *IDH*-WT GBM. *ATRX* has been implicated in several roles including CR and regulating telomere length, but has also been independently associated with poor survival and loss of genomic stability, including impairment of nonhomologous end-joining DNA repair.^28,29^ *ATRX*-deficient mouse models have exhibited sensitivity to DNA damaging agents,^28^ and *ATRX*-mutated tumors may be explored as targets for other therapies such as PARP inhibition.

The prognostic value of CR genes was observed by Kaplan–Meier analysis, with an observed median OS difference of approximately 2 months (*P* = .004) between CR-mut and CR-WT GBM, *IDH*-WT (Figure 5).

When multiple comparisons are undertaken in an exploratory study of this type, the risk of a type I error is increased. We have attempted to address this risk by using appropriate statistical techniques (Bonferroni and Benjamini-Hochberg) as appropriate. While our study cohorts were stratified by *IDH* status, we performed multivariate analysis to attempt to control for additional potential confounders including age, *MGMT* methylation, TMZ treatment, and chromosomal abnormalities commonly found in glioma. Younger age, *MGMT* methylation, and TMZ treatment were favorable factors associated with increased survival in our GBM, *IDH*-WT model, thus confirming previous observations in HGGs. However, after accounting for these additional covariants, the significant negative association between CR-mutated tumors and OS in GBM, *IDH*-WT remains.

The divergence of survival outcomes in the GBM, *IDH* wild-type, and astrocytoma, *IDH-*mutated cohorts is perhaps unexpected and may merit some comment. Low patient numbers in the astrocytoma *IDH*-mutated cohort is one possible explanation; however, increasing the sample size typically does not reverse the direction of an effect, but only provides a more precise estimate.

In conclusion, the most significant finding in our study was the association between CR-mut genes and unfavorable OS in *IDH*-WT glioblastoma. Our study also points to several biomarker candidates, both positive and negative, for other emerging therapeutic strategies. The complexity of involvement in CR genes in oncogenic networks demands a better understanding of some of these mechanisms in order to translate emerging strategies into successful applications in HGG.

## References

[1] Molinaro AM, Taylor JW, Wiencke JK, Wrensch MR. Genetic and molecular epidemiology of adult diffuse glioma. Nat Rev Neurol. 2019;15(7):405–417. 31227792 10.1038/s41582-019-0220-2 PMC7286557

[2] Louis DN, Perry A, Wesseling P, et al The 2021 WHO classification of tumors of the central nervous system: A summary. Neuro Oncol. 2021;23(8):1231–1251. 34185076 10.1093/neuonc/noab106 PMC8328013

[3] Fisher JP, Adamson DC. Current FDA-approved therapies for high-grade malignant gliomas. Biomedicines. 2021;9(3):324. 33810154 10.3390/biomedicines9030324 PMC8004675

[4] Centore RC, Sandoval GJ, Soares LMM, Kadoch C, Chan HM. Mammalian SWI/SNF chromatin remodeling complexes: Emerging mechanisms and therapeutic strategies. Trends Genet. 2020;36(12):936–950. 32873422 10.1016/j.tig.2020.07.011

[5] Ribeiro-Silva C, Vermeulen W, Lans H. SWI/SNF: Complex complexes in genome stability and cancer. DNA Repair (Amst). 2019;77(May):87–95. 30897376 10.1016/j.dnarep.2019.03.007

[6] Abakir A, Ruzov A. SWI/SNF complexes as determinants of R-loop metabolism. Nat Genet. 2021;53(7):940–941. 34244698 10.1038/s41588-021-00884-1

[7] Valor LM, Hervás-Corpión I. The epigenetics of glioma stem cells: A brief overview. Front Oncol. 2020;10(Dec):602378. 33344253 10.3389/fonc.2020.602378 PMC7738619

[8] McClellan BL, Haase S, Nunez FJ, et al Impact of epigenetic reprogramming on antitumor immune responses in glioma. J Clin Invest. 2023;133(2):e163450. 36647827 10.1172/JCI163450 PMC9843056

[9] Finotello F, Mayer C, Plattner C, et al Molecular and pharmacological modulators of the tumor immune contexture revealed by deconvolution of RNA-seq data. Genome Med. 2019;11(1):34. 31126321 10.1186/s13073-019-0638-6 PMC6534875

[10] Marabelle A, Fakih M, Lopez J, et al Association of tumour mutational burden with outcomes in patients with advanced solid tumours treated with pembrolizumab: Prospective biomarker analysis of the multicohort, open-label, phase 2 KEYNOTE-158 study. Lancet Oncol. 2020;21(10):1353–1365. 32919526 10.1016/S1470-2045(20)30445-9

[11] Marabelle A, Cassier PA, Fakih M, et al Pembrolizumab for previously treated advanced anal squamous cell carcinoma: Results from the non-randomised, multicohort, multicentre, phase 2 KEYNOTE-158 study. Lancet Gastroenterol Hepatol. 2022;7(5):446–454. 35114169 10.1016/S2468-1253(21)00382-4 PMC12012850

[12] Goldstein M, Gabriel N, Inkman M, Zhang J, Dahiya S. DDRE-32. SETD2 histone methyltransferase mutation status predicts treatment response in glioblastoma: Strategies to overcome chemoresistance. Neuro-Oncology. 2021;23(suppl_6):vi81–vi81.

[13] Wanior M, Krämer A, Knapp S, Joerger AC. Exploiting vulnerabilities of SWI/SNF chromatin remodelling complexes for cancer therapy. Oncogene. 2021;40(21):3637–3654. 33941852 10.1038/s41388-021-01781-x PMC8154588

[14] Mandal J, Mandal P, Wang TL, Shih IM. Treating ARID1A mutated cancers by harnessing synthetic lethality and DNA damage response. J Biomed Sci. 2022;29(1):71. 36123603 10.1186/s12929-022-00856-5 PMC9484255

[15] Williamson CT, Miller R, Pemberton HN, et al ATR inhibitors as a synthetic lethal therapy for tumours deficient in ARID1A. Nat Commun. 2016;7(Dec):13837. 27958275 10.1038/ncomms13837 PMC5159945

[16] Srinivas US, Tay NSC, Jaynes P, et al PLK1 inhibition selectively induces apoptosis in ARID1A deficient cells through uncoupling of oxygen consumption from ATP production. Oncogene. 2022;41(13):1986–2002. 35236967 10.1038/s41388-022-02219-8

[17] Liu N, Hu G, Wang H, Li Z, Guo Z. PLK1 inhibitor facilitates the suppressing effect of temozolomide on human brain glioma stem cells. J Cell Mol Med. 2018;22(11):5300–5310. 30133120 10.1111/jcmm.13793 PMC6201353

[18] Shi H, Sun S, Xu H, et al Combined delivery of temozolomide and siPLK1 using targeted nanoparticles to enhance temozolomide sensitivity in glioma. Int J Nanomedicine. 2020;15(May):3347–3362. 32494134 10.2147/IJN.S243878 PMC7229804

[19] Li Y, Yang X, Zhu W, et al SWI/SNF complex gene variations are associated with a higher tumor mutational burden and a better response to immune checkpoint inhibitor treatment: A pan-cancer analysis of next-generation sequencing data corresponding to 4591 cases. Cancer Cell Int. 2022;22(1):347. 36371186 10.1186/s12935-022-02757-x PMC9652899

[20] Wang D, Elenbaas B, Murugesan K, et al Relationship among DDR gene mutations, TMB and PD-L1 in solid tumour genomes identified using clinically actionable biomarker assays. npj Precis Oncol. 2023;7(1):103. 37821580 10.1038/s41698-023-00442-4 PMC10567713

[21] Zhu Y, Yan C, Wang X, et al Pan-cancer analysis of ARID family members as novel biomarkers for immune checkpoint inhibitor therapy. Cancer Biol Ther. 2022;23(1):104–111. 35239432 10.1080/15384047.2021.2011643 PMC8896200

[22] Zhou M, Yuan J, Deng Y, Fan X, Shen J. Emerging role of SWI/SNF complex deficiency as a target of immune checkpoint blockade in human cancers. Oncogenesis. 2021;10(1):3. 33419967 10.1038/s41389-020-00296-6 PMC7794300

[23] AACR Project GENIE: Powering precision medicine through an International Consortium. Cancer Discov. 2017;7(8):818–831. 28572459 10.1158/2159-8290.CD-17-0151 PMC5611790

[24] Jin Y, Xiao W, Song T, Feng G, Dai Z. Expression and prognostic significance of p53 in glioma patients: A meta-analysis. Neurochem Res. 2016;41(7):1723–1731. 27038932 10.1007/s11064-016-1888-y

[25] Li J, Liang R, Song C, Xiang Y, Liu Y. Prognostic significance of epidermal growth factor receptor expression in glioma patients. Onco Targets Ther. 2018;11(Feb):731–742. 29445288 10.2147/OTT.S155160 PMC5808691

[26] Aquilanti E, Miller J, Santagata S, Cahill DP, Brastianos PK. Updates in prognostic markers for gliomas. Neuro Oncol. 2018;20(suppl_7):viivii17–viivii26. 10.1093/neuonc/noy158 PMC6225747 30412261

[27] Yang K, Wu Z, Zhang H, et al Glioma targeted therapy: Insight into future of molecular approaches. Mol Cancer. 2022;21(1):39. 35135556 10.1186/s12943-022-01513-z PMC8822752

[28] Koschmann C, Calinescu AA, Nunez FJ, et al ATRX loss promotes tumor growth and impairs nonhomologous end joining DNA repair in glioma. Sci Transl Med. 2016;8(328):328ra328. 10.1126/scitranslmed.aac8228 PMC5381643 26936505

[29] Koschmann C, Lowenstein PR, Castro MG. ATRX mutations and glioblastoma: Impaired DNA damage repair, alternative lengthening of telomeres, and genetic instability. Mol Cell Oncol. 2016;3(3):e1167158. 27314101 10.1080/23723556.2016.1167158 PMC4909411

